# Longitudinal assessment of health-related quality of life in Japanese patients with advanced urothelial carcinoma receiving immune check point inhibitors

**DOI:** 10.1038/s41598-024-72755-8

**Published:** 2024-10-04

**Authors:** Makito Miyake, Nobutaka Nishimura, Yuki Oda, Tatsuki Miyamoto, Kota Iida, Mitsuru Tomizawa, Takuto Shimizu, Takuya Owari, Kenta Ohnishi, Shunta Hori, Yosuke Morizawa, Daisuke Gotoh, Yasushi Nakai, Kazumasa Torimoto, Tomomi Fujii, Nobumichi Tanaka, Kiyohide Fujimoto

**Affiliations:** 1 Department of Urology, Nara Medical University, 840 Shijo-cho, Kashihara, Nara 634-8522 Japan; 2 Division of Fostering Required Medical Human Resources, Center for Infectious Disease Education and Research (CiDER), Osaka University, Osaka, 565-0871 Japan; 3 Department of Diagnostic Pathology, Nara Medical University, Kashihara, Nara 634-8522 Japan; 4 Department of Prostate Brachytherapy, Nara Medical University, Kashihara, Nara 634-8522 Japan

**Keywords:** Immune checkpoint inhibitors, Patient reported outcome measures, Platinum, Quality of life, Urinary bladder neoplasms, Urothelial carcinoma, Urology, Bladder, Ureter, Urogenital diseases

## Abstract

Real-world data on health-related quality of life (HRQoL) in advanced urothelial carcinoma (aUC) receiving immune checkpoint inhibitors (ICIs) are limited. This study included 42 patients with aUC who received second-line or later pembrolizumab (*n* = 19), maintenance avelumab followed by first-line chemotherapy (*n* = 13), or adjuvant nivolumab after radical surgery (*n* = 10). Time-course changes in the domains and scales related to HRQoL were evaluated using the EORTC QLQ-C30, FACT-G, and SF-8 questionnaires during ICI therapy. Anchor-based approaches for minimally important differences were determined as ‘improved’, ‘stable’, and ‘deteriorated’. We found significant improvements after the start of pembrolizumab treatment on many scales. Almost none of the scales changed significantly in the avelumab and nivolumab groups. Approximately 80% of the pembrolizumab group had deteriorated social/family well-being in FACT-G. Approximately 60% of the patients in the avelumab group had deteriorated general health and vitality in SF-8. In the nivolumab group, none of the scales deteriorated in > 50% of the patients. Deterioration of physical function in the SF-8 was associated with occurrence of treatment-related adverse events ≥ grade 2 during ICI therapy (*P* = 0.013). Our findings demonstrated that majority of patients with aUC who received ICI therapy had a stable HRQoL, which was consistent with evidence from clinical trials.

## Introduction

Urothelial carcinoma (UC) develops in the mucosa of the renal pelvis, ureters, bladder, or urethra. Regarding bladder UC, approximately 70–80% of newly diagnosed patients have non-muscle invasive disease, while the remaining 20–30% have advanced UC (aUC) with or without unresectable or metastatic lesions^1^. Despite the recent advancements in therapeutic modalities, aUC remains associated with poor survival outcomes. This disease subset requires multidisciplinary management, including surgery, radiotherapy, and systemic therapy using platinum-based chemotherapy, immune checkpoint inhibitors (ICIs), taxane-based chemotherapy, enfortumab vedotin, and fibroblast growth factor receptor-targeted therapy^2^. In Japan, three ICIs, second-line or later pembrolizumab for unresectable/metastatic UC (mUC) (2017), maintenance avelumab for mUC (2021), and adjuvant nivolumab for muscle-invasive UC (MIUC) (2022), have been approved based on the positive results of the KEYNOTE-045^3^, JAVELIN Bladder 100^4^, and CheckMate-274 trials^5^, respectively. Owing to the heterogeneity in tumor response and tolerability to ICI therapy, treatment duration varies significantly among patients.

Particularly in aUC, bothersome symptoms such as pain, hematuria, dysuria, urinary obstruction, constipation, and emotional distress affect the health-related quality of life (HRQoL). Recently, the measurement of patient-reported outcomes (PROs) has been considered a vital aspect of cancer care^6^. Cancer treatment-related physical and psychological changes affect daily activities^7,8^. Taarnhøj et al. reported that psychological issues have a strong impact on HRQoL; therefore, this should be managed properly during chemo- or immunotherapy to maintain the best possible QoL in patients with aUC of the bladder^9^. The KEYNOTE-045^10,11^, JAVELIN Bladder 100^12^, and CheckMate-274 trials^13,14^ have released sub-analysis data regarding the effects of ICI therapy on HRQoL.

The European Association of Urology guidelines on muscle-invasive and metastatic bladder cancer strongly recommend the use of validated questionnaires to assess HRQoL in patients with muscle-invasive bladder cancer, both at baseline and after treatment^2^. However, real-world data on the HRQoL of patients with aUC treated with ICIs are sparse, particularly in Japan. Emerging therapies for aUC have accelerated interest in accumulating evidence of PRO measurements in this patient population to capture meaningful changes in HRQOL. Herein, we performed an ambispective clinical study on a time-course assessment using validated questionnaires to describe the changes in HRQoL during ICI therapy in patients with aUC.

## Patients and methods

### Patient enrollment and data collection

This single-center prospective study was approved by the Ethics Committee of Nara Medical University (protocol ID: NMU-1719) and the study protocol complied with the Declaration of Helsinki (2013). This clinical study was registered on 28/03/2024 with the Japan Registry of Clinical Trials (jRCT1051230211), which is a publically accessible primary register that participates in the WHO International Clinical Trial Registry Platform (https://www.who.int/clinical-trials-registry-platform/network/primary-registries). This study aimed to evaluate the time-course changes in the domains and functions of the European Organization for Research and Treatment of Cancer Quality of Life Questionnaire-Core 30 (EORTC QLQ-C30), Functional Assessment of Cancer Therapy–General (FACT-G), and multi-item short form-8 (SF-8) during ICI therapy.

This study included 42 patients with aUC who received at least one of the following intravenous systemic ICIs between March 2018 and July 2023: second-line or later pembrolizumab, maintenance avelumab followed by first-line chemotherapy, or adjuvant nivolumab following radical surgery. Other key eligibility criteria were pathologically confirmed UC and a willingness to adhere to the study protocol. Written informed consent was obtained from all the participants. We recorded the patients’ baseline characteristics including age, sex, Eastern Cooperative Oncology Group performance status (PS), smoking history, estimated glomerular filtration rate (eGFR), primary tumor origin, radical surgery, and unresectable/metastatic lesions.

### Dose of ICI therapies

Pembrolizumab was administered at a dose of 200 mg every three weeks or 400 mg every six weeks. Maintenance avelumab was administered at a dose of 10 mg/kg every two weeks. Adjuvant nivolumab was administered at a dose of 480 mg every month for up to one year. Dose intervals, dose interruptions, and setting of drug holidays depended on the physician’s decision.

### Assessment of patient-reported HRQoL

PROs related to HRQoL were assessed using three questionnaires: EORTC QLQ-C30^15,16^, FACT-G^17^, and SF-8^18^. Participants completed all questionnaires at baseline and once a month during treatment. The analysis included baseline and all-on-treatment assessments, excluding the end-of-treatment assessments. Global health status/QoL and five functional scales were calculated according to a scoring procedure^19^. Higher scores on a functional scale and global health status/QoL indicate better QoL. The FACT-G consists of 27 items grouped into four domains: physical well-being, social/family well-being (SWB), emotional well-being (EWB), and functional well-being (FWB). The score was calculated according to the FACT-G scoring guidelines, and the FACT-G total score consisted of the sum of the four subdomains, ranging from 0 to 108. Higher scores on a functional scale and global health status/QoL indicate better QoL. The SF-8 contains psychometrically based physical and mental health summary measures, scoring eight domains, and two component summaries, which are calculated by weighing each SF-8 item using a norm-based scoring method given in the instrument guidelines^20^. Higher domain scores, physical component summary (PCS), and mental component summary (MCS), indicate better health status. Scores above and below 50 are considered above and below average, respectively, in the general U.S. population^20^.

Baseline scores were obtained on day 1 after the initial ICI therapy. The cut-off for the minimally important difference (MID) of each scale was defined as 10 points on the EORTC QLQ-C30^10,11^ and half the standard deviation (SD) of the baseline score on the FACT-G and SF-8^21^. Anchor-based approaches to determine MID were as follows: Increase ≥ MID = ‘improved,’ decrease ≥ MID = ‘deteriorated’ from baseline score prior to the end of treatment, and otherwise ‘stable.’

#### Assessment of adverse events

Adverse events (AEs) observed during treatment were graded according to the National Cancer Institute Common Terminology Criteria for Adverse Events version 5.0. All treatment-related AEs (TRAEs) were determined by the investigators based on their potential immunological etiology. Because the AEs were evaluated based on a chart review, it was difficult to record grade 1 AEs; therefore, AEs ≥ grade 2 were recorded.

### Statistical analysis

All recorded values were tabulated and graphically plotted. The HRQoL scales at each time point were compared with those at baseline using the Wilcoxon signed-rank test. The overall time course changes in each scale at baseline and during treatment were assessed using linear mixed-effects models for repeated measures. For each model, the respective scores were used as fixed effects and the intercept and time (treated as a continuous variable) were used as random effects. Fisher’s exact test was used to evaluate potential association between occurrence of TRAEs ≥ grade 2 and change in scales (‘improved/stable’ or ‘deteriorated’) of HRQoL. PRISM software, version 9.5.1 (GraphPad Software, Inc., San Diego, CA, USA) was used for illustrations, and EZR (version 4.3.1) was used for statistical analysis^22^. A *P* value < 0.05 was considered statistically significant.

## Results

This study included 19 patients who received pembrolizumab (Pem group), 13 who received maintenance avelumab (Ave group), and 10 who received adjuvant nivolumab (Nivo group). The baseline characteristics of the 42 patients are presented in Table 1. Most patients had an ECOG-PS score of 0 or 1. The Nivo group was associated with older age, lower eGFR, and a higher rate of patients with upper urinary tract cancer. Median duration of treatment was 9 (range, 2−16), 4 (range, 3−14), and 12 (range, 2−12) months in the Pem, Ave, and Nivo groups, respectively. The overall and per-assessment completion rates of the three questionnaires at each assessment point were > 90% during the treatment period. The proportion of eligible patients who completed the questionnaires decreased over time within 10 months after the treatment initiation in the three arms. The reasons for discontinuation of questionnaire survey are shown in Supplementary Table S1. The major reasons were discontinuation of treatment due to progression or intolerable adverse events and patient preference to stop questionnaire survey.

**Table 1:** Baseline clinicopathologic variables of patients with advanced urothelial carcinoma who received immune checkpoint inhibitors.

Variables	Overall	Second-line or later pembrolizumab	Maintenance avelumab	Adjuvant nivolumab
Total, n	42	19	13	10
Age, years, mean ± SD	74.6 ± 8.7	71.1 ± 10.2	75.6 ± 6.1	80.0 ± 5.1
Sex				
Male	31 (74%)	16 (84%)	10 (77%)	5 (50%)
Female	11 (26%)	3 (16%)	3 (23%)	5 (50%)
ECOG-PS				
0	33 (79%)	16 (84%)	12 (92%)	5 (50%)
1	8 (19%)	3 (16%)	1 (7.7%)	4 (40%)
2	1 (2.4%)	0	0	1 (10%)
Smoking				
Never	13 (31%)	4 (21%)	5 (39%)	4 (40%)
Former	20 (48%)	11 (58%)	4 (31%)	5 (50%)
Current	6 (14%)	3 (16%)	3 (23%)	0
Unknown	3 (7.1%)	1 (5.3%)	1 (7.7%)	1 (10%)
eGFR, mL/min/1.73m2, mean ± SD	47.1 ± 18.7	50.2 ± 12.7	52.0 ± 25.8	35.9 ± 10.4
Primary disease				
Bladder	21 (50%)	10 (53%)	8 (62%)	3 (30%)
Renal pelvis	14 (33%)	6 (32%)	4 (31%)	4 (40%)
Ureter	7 (17%)	3 (16%)	1 (7.7%)	3 (30%)
Radical surgery				
Cystectomy	14 (33%)	6 (32%)	5 (39%)	3 (30%)
Nephroureterectomy	17 (41%)	7 (37%)	3 (23%)	7 (70%)
No	11 (26%)	6 (32%)	5 (39%)	0
Unresectable/metastatis lesions^#^				
Primary disease	8 (19%)	6 (32%)	2 (15%)	0
Local recurrence arround primary disease	8 (19%)	2 (11%)	6 (46%)	0
Lymph nodes	21 (50%)	13 (68%)	8 (62%)	0
Lung	10 (24%)	6 (32%)	4 (31%)	0
Liver	3 (7.1%)	1 (5.3%)	2 (15%)	0
Bone	6 (14%)	5 (26%)	1 (7.7%)	0
Peritonium	1 (2.4%)	0	1 (7.7%)	0

*SD* standard deviation, *ECOG-PS* Eastern cooperative oncology group performance status, *eGFR* estimated glomerular filtration rate; ^#^ Some patients had multiple lesions.

**Fig. 1:**
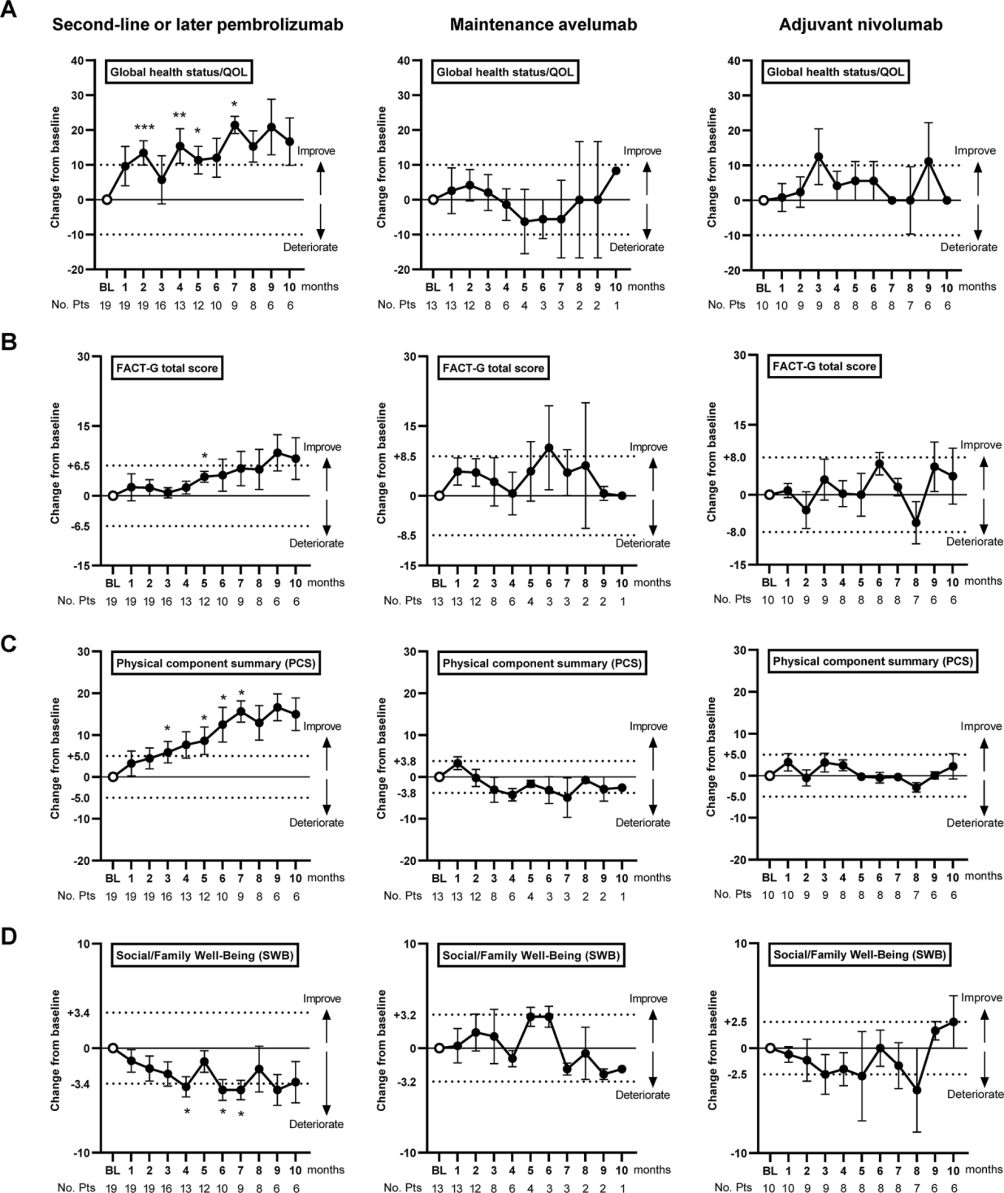
Mean changes in scales during immune checkpoint inhibitor (ICI) therapy for advanced urothelial carcinoma. Data are expressed as means ± standard deviations. A higher score indicates a higher quality of life. Each score during treatment was compared to ‘before the initial dose of ICI (baseline, BL)’ using Wilcoxon signed-rank test (*P < 0.05, **P < 0.01, and ***P < 0.001). Dashed lines indicate the minimally important difference (MIC) for each scale. Global health status/QoL was assessed using the European Organization for Research and Treatment of Cancer Quality of Life Questionnaire-Core 30 (**A**). The functional assessment of cancer therapy-general (FACT-G) questionnaire was used to calculate total FACT-G scores (**B**) and social/family well-being (**D**). The physical component summary (PCS) was based on multi-item short form-8 questionnaire (**C**).

The baseline scores of the domains and functional scales of HRQoL, MIDs, and the estimated mean change per month are listed according to the administered ICI in Table 2. At baseline (before the start of ICI therapy), global health status/QoL in the EORTC QLQ-C30 was 54 ± 21 (mean ± SD), 62 ± 26, and 63 ± 19 in the Pem, Ave, and Nivo groups, respectively. Global health status/QoL improved significantly over time in the Pem group, but no significant changes were noted in the other two groups (Fig. 1A). Similar results were observed for other domains and functional scales, including the FACT-G total score and PCS (Table 1; Fig. 1B−D). In contrast, pembrolizumab and avelumab negatively affected SWB in the FACT-G questionnaire (estimated mean change, -0.27; *P* = 0.017 and estimated mean change, -0.43; *P* = 0.056, respectively); however, no significant effect was observed in the Nivo group (0.26; *P* = 0.15).

**Table 2:** Linear mixed-effects model for repeated measures (MMRM) of the domains and functional scales of health-related quality of life questionnaires in patients with advanced urothelial carcinoma treated with immune checkpoint inhibitors.

Questionnaire	Domains and functions (abbreviation)	Second-line or later pembrolizumab (*n* = 19)				Maintenance avelumab (*n* = 13)				Adjuvant nivolumab (*n* = 10)			
		Baseline	MID_#_	Estimated mean change per a month (standard error)	*P* value^##^	Baseline	MID^#^	Estimated mean change per a month (standard error)	*P* value^##^	Baseline	MID^#^	Estimated mean change per a month (standard error)	*P* value^##^
EORTC QLQ-C30													
	Global health status/QoL	54 ± 21	± 10	1.31 (0.56)	0.022	62 ± 26	± 10	-0.63 (0.94)	0.51	63 ± 19	± 10	0.27 (0.60)	0.66
	Physical functioning (PF)	72 ± 24	± 10	1.02 (0.41)	0.015	77 ± 18	± 10	0.20 (0.59)	0.73	74 ± 20	± 10	0.50 (0.37)	0.18
	Role functioning (RF)	72 ± 24	± 10	1.18 (0.43)	0.007	83 ± 16	± 10	-0.15 (0.48)	0.76	83 ± 18	± 10	0.12 (0.31)	0.69
	Emotional functioning (EF)	75 ± 24	± 10	0.81 (0.43)	0.06	81 ± 20	± 10	-0.34 (0.37)	0.36	79 ± 23	± 10	0.57 (0.47)	0.23
	Cognitive functioning (CF)	72 ± 24	± 10	0.25 (0.44)	0.56	72 ± 18	± 10	0.34 (0.57)	0.56	77 ± 24	± 10	0.44 (0.58)	0.46
	Social functioning (SF)	82 ± 21	± 10	-0.62 (0.47)	0.19	85 ± 20	± 10	-0.85 (0.62)	0.18	78 ± 22	± 10	0.42 (0.26)	0.11
FACT-G													
	Physical well-being (PWB)	22 ± 5.5	± 2.8	0.28 (0.10)	0.005	22 ± 4.3	± 2.2	0.09 (0.11)	0.42	21 ± 4.7	± 2.4	0.29 (0.11)	0.012
	Social/family well-being (SWB)	14 ± 6.7	± 3.4	-0.27 (0.11)	0.017	12 ± 6.3	± 3.2	-0.43 (0.22)	0.056	13 ± 4.9	± 2.5	0.26 (0.18)	0.15
	Emotional well-being (EWB)	16 ± 4.5	± 2.3	0.14 (0.09)	0.120	17 ± 4.1	± 2.2	0.35 (0.15)	0.019	18 ± 4.7	± 2.4	0.26 (0.14)	0.064
	Functional well-being (FWB)	16 ± 5.5	± 2.8	0.39 (0.13)	0.005	14 ± 8.8	± 4.4	-0.82 (0.32)	0.014	16 ± 6.9	± 3.5	-0.19 (0.19)	0.32
	FACT-G total score	68 ± 13	± 6.5	0.56 (0.22)	0.011	64 ± 17	± 8.5	-0.94 (0.44)	0.14	68 ± 16	± 8.0	0.53 (0.35)	0.14
SF-8													
	Physical function (PF)	41 ± 12	± 6.0	0.52 (0.20)	0.011	45 ± 7.6	± 3.8	-0.19 (0.28)	0.51	42 ± 12	± 6.0	-0.16 (0.20)	0.43
	Role physical (RP)	41 ± 11 0.59 (0.21)	± 5.5		0.007	46 ± 8.5	± 4.3	-0.32 (0.26)	0.22	43 ± 13	± 6.5	0.09 (0.17)	0.58
	Bodily pain (BP)	48 ± 10	± 5.0	0.71 (0.23)	0.002	56 ± 7.6	± 3.8	-0.59 (0.27)	0.34	50 ± 7.5	± 3.8	-0.02 (0.29)	0.95
	General health (GH)	45 ± 8.0	± 4.0	0.68 (0.19)	< 0.001	51 ± 8.0	± 4.0	-0.24 (0.27)	0.39	48 ± 6.9	± 3.5	0.03 (0.15)	0.86
	Vitality (VT)	47 ± 7.2	± 3.6	0.55 (0.17)	0.002	51 ± 8.3	± 4.2	-0.48 (0.25)	0.061	48 ± 7.8	± 3.9	0.37 (0.16)	0.035
	Social functioning (SF)	44 ± 11	± 5.5	-0.62 (0.47)	0.16	50 ± 9.1	± 4.6	-0.43 (0.27)	0.077	44 ± 13	± 6.5	-0.10 (0.21)	0.63
	Role emotional (RE)	43 ± 9.2	± 4.6	0.44 (0.17)	0.009	49 ± 7.7	± 3.9	-0.51 (0.28)	0.28	45 ± 8.7	± 4.4	0.01 (0.19)	0.93
	Mental health (MH)	46 ± 8.0	± 4.0	0.50 (0.15)	0.001	52 ± 6.9	± 3.5	-0.40 (0.22)	0.078	47 ± 9.4	± 4.7	0.10 (0.18)	0.60
	Physical component summary (PCS)	42 ± 10	± 5.0	0.64 (0.21)	0.003	47 ± 7.5	± 3.8	-0.34 (0.25)	0.18	44 ± 10	± 5.0	-0.04 (0.17)	0.82
	Mental component summary (MCS)	46 ± 8.2	± 4.1	0.32 (0.16)	0.055	50 ± 6.4	± 3.2	-0.33 (0.25)	0.18	46 ± 7.8	± 3.9	0.12 (0.18)	0.51

Data are expressed by means ± standard deviations. A higher score indicates a higher level of quality of life.

*EORTC QLQ-C30* the European organization for research and treatment of cancer quality of life questionnaire - core 30, *FACT-G* functional assessment of cancer therapy-general, *SF-8* 8-Item short form survey, *MID* minimally important difference.

^#^Minimally important difference (MID) of each scale are defined ± 10 points in the EORTC QLQ-C30, and half a standard deviation of the baseline score in the FACT-G and SF-8 prior to the end of treatment.

^##^The linear mixed-effects model for repeated measures was used to detect statistically significant changes during the treatment. groups.

To explore possible association of improvement on HRQoL with objective response to pembrolizumab in the Pem group, we compared time-course changes of HRQoL between patient showing complete response (CR) or partial response (PR) and those showing stable disease (SD) or progressive disease (PD) according to the response evaluation criteria in solid tumors (RESCIST) v1.1. The objective response was CR in one, PR in six, SD in seven, and PD in five out of 19 patients in the Pem group, respectively. Supplementary Figure S1 demonstrates the difference of time-course change of global health status/QoL between CR/PR patients and SD/PD patients, suggesting a trend of better QoL in CR/PR patients as compared to SD/PD patients, but that did not reach statistical significance due to the small number of cases.

**Fig. 2:**
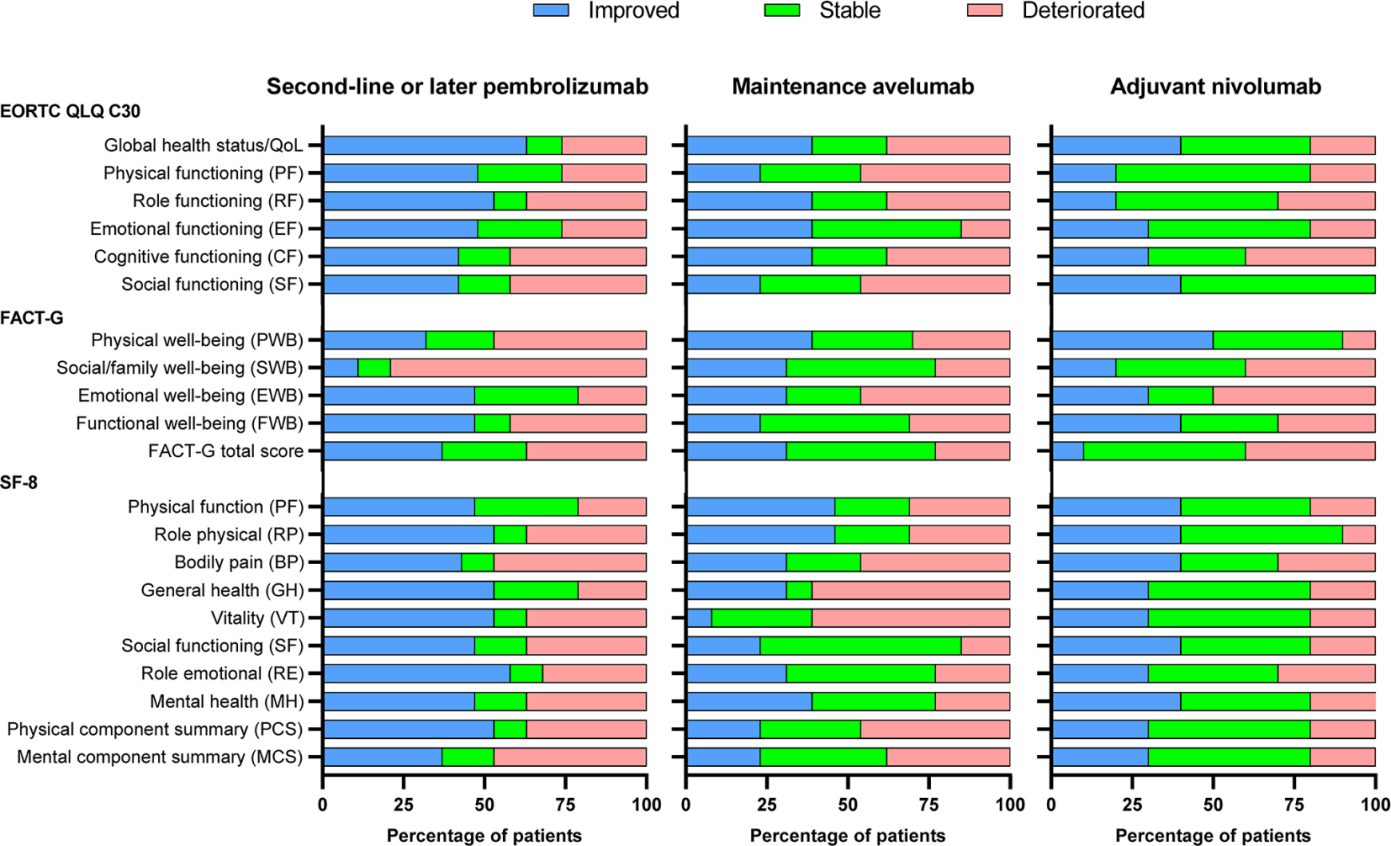
Proportion of patients with improved, stable, and deteriorated domains and functional scales during immune checkpoint inhibitor (ICI) therapy. Baseline scores were obtained on day 1 after the initial ICI therapy. The minimally important difference (MIC) of each scale was defined as 10 points in the EORTC QLQ-C30 and half the standard deviation (SD) of the baseline score in the FACT-G and SF-8. Increased and decreased ≥ MIC from the baseline score during ICI therapy were considered ‘improved’ and ‘deteriorated’, respectively. EORTC QLQ-C30, European organization for research and treatment of cancer quality of life questionnaire-core 30; *FACT-G*, functional assessment of cancer therapy-general; *SF-8* multi-item short form-8.

Patients were categorized into three types according to the change of domains and functional scales during ICI therapy: ‘improved,’ ‘stable,’ and ‘deteriorated.’ Fig. 2 shows the distribution of the patients using bar plots. In general, the distribution varied among the Pem, Ave, and Nivo groups. Notably, approximately 80% of the patients in the Pem group had deteriorated SWB, but other domains and scales were not deteriorated in > 50% of the patients in the Pem group. Moreover, approximately 60% of the patients in the Ave group had deteriorated general health and vitality in the SF-8 survey. In the Nivo group, none of the domains or scales deteriorated in more than half of the patients. 

Lastly, we investigated whether change in each scale (‘improved/stable’ or ‘deteriorated’) of HRQoL were associated with ccurrence of  TRAEs ≥ grade 2 . Of the 42 patients who received ICI therapy, 23 (55%) experienced TRAEs ≥ grade 2 and 4 (9.5%) experienced TRAEs ≥ grade 3 (Table 3). Nine (47%) of the 19 patients in the Pem group, 7 (54%) of the 13 in the Ave group, and 7 (70%) of the 10 in the Nivo group had TRAEs ≥ grade 2. Table 4 lists 2 × 2 contingency tables for the association between the deterioration of each scale and the occurrence of TRAEs ≥ grade 2 in 42 patients. Deterioration of physical function in the SF-8 was statistically associated with the occurrence of TRAEs ≥ grade 2 during ICI therapy. 

**Table 3:** Treatment-related adverse events during treatment with immune checkpoint inhibitors.

Treatment-related adverse events ^#^	Overall (*n* = 42)		Pembrolizumab (*n* = 19)		Maintenance avelumab (*n* = 13)		Adjuvant nivolumab (*n* = 10)	
	Grade 2 ≤	Grade 3 ≤	Grade 2 ≤	Grade 3 ≤	Grade 2 ≤	Grade 3 ≤	Grade 2 ≤	Grade 3 ≤
Any ^##^	23 (55%)	4 (9.5%)	9 (47%)	2 (11%)	7 (54%)	1 (7.7%)	7 (70%)	0
Myelosuppression	1 (2.4%)	0	0	0	1 (7.7%)	0	0	0
Gastrointestinal disorder	3 (7.1%)	0	1 (5.3%)	0	1 (7.7%)	0	1 (10%)	0
Fatigue	1 (2.4%)	1 (2.4%)	1 (5.3%)	1 (5.3%)	0	0	0	0
Hepatotoxicity	2 (4.8%)	0	2 (11%)	0	0	0	0	0
Dermatoxicity	7 (17%)	1 (2.4%)	2 (11%)	0	4 (31%)	1 (7.7%)	1 (10%)	0
Interstitial lung disease	4 (9.5%)	0	3 (16%)	0	0	0	1 (10%)	0
Colitis	2 (4.8%)	0	1 (5.3%)	0	0	0	1 (10%)	0
Endocrine disorders	10 (24%)	2 (4.8%)	5 (26%)	1 (5.3%)	1 (7.7%)	0	4 (40%)	1 (10%)
Type 1 diabetes	1 (2.4%)	0	0	0	1 (7.7%)	0	0	0

No patients had grade 4 ≤ adverse events during the treatment.

^#^ Determined by the investigator to be drug related; ^##^ A patient with multiple adverse events is counted one.

**Table 4:** Contingency tables showing the association between deterioration on each scale and the occurrence of grade 2 £ treatment-related adverse events.

Questionnaire	Domains and functions (abbreviation)	Change during ICI therapy	Total	Grade 2 ≤ adverse events		Odds ratio (95% confidence interval)	Fisher’s exact test *P* value
				No	Yes		
Total			42	19	23		
EORTC QLQ-C30	Global health status/QoL	Improved/stable	30	16	14		
		Deteriorated	12	3	9	3.5 (0.77–12.2)	0.17
	Physical functioning (PF)	Improved/stable	29	15	14		
		Deteriorated	13	4	9	2.4 (0.61–8.2)	0.32
	Role functioning (RF)	Improved/stable	27	13	14		
		Deteriorated	15	6	9	1.4 (0.40–4.5)	0.75
	Emotional functioning (EF)	Improved/stable	33	15	18		
		Deteriorated	9	4	5	1.0 (0.23–3.9)	0.99
	Cognitive functioning (CF)	Improved/stable	25	13	12		
		Deteriorated	17	6	11	2.0 (0.60–6.3)	0.35
	Social functioning (SF)	Improved/stable	27	12	15		
		Deteriorated	15	7	8	0.91 (0.26–3.4)	0.99
FACT-G	Physical well-being (PWB)	Improved/stable	28	13	15		
		Deteriorated	14	6	8	1.2 (0.31–3.8)	0.99
	Social/family well-being (SWB)	Improved/stable	20	7	13		
		Deteriorated	22	12	10	0.45 (0.12–1.4)	0.23
	Emotional well-being (EWB)	Improved/stable	27	14	13		
		Deteriorated	15	5	10	2.1 (0.61–7.7)	0.34
	Functional well-being (FWB)	Improved/stable	27	12	15		
		Deteriorated	15	7	8	0.91 (0.26–3.4)	0.99
	FACT-G total score	Improved/stable	28	12	16		
		Deteriorated	14	7	7	0.75 (0.19–2.9)	0.75
SF-8	Physical function (PF)	Improved/stable	32	18	14		
		Deteriorated	10	1	9	11.6 (1.7–133)	0.013
	Role physical (RP)	Improved/stable	30	16	14		
		Deteriorated	12	3	9	3.4 (0.77–13.2)	0.17
	Bodily pain (BP)	Improved/stable	24	12	12		
		Deteriorated	18	7	11	1.6 (0.49–5.7)	0.54
	General health (GH)	Improved/stable	28	14	15		
		Deteriorated	14	6	8	1.2 (0.35–4.1)	0.99
	Vitality (VT)	Improved/stable	25	12	13		
		Deteriorated	17	7	10	1.3 (0.40–4.8)	0.76
	Social functioning (SF)	Improved/stable	31	15	16		
		Deteriorated	11	4	7	1.6 (0.36–5.8)	0.73
	Role emotional (RE)	Improved/stable	30	14	16		
		Deteriorated	12	5	7	1.2 (0.29–4.6)	0.99
	Mental health (MH)	Improved/stable	30	14	16		
		Deteriorated	12	5	7	1.2 (0.29–4.6)	0.99
	Physical component summary (PCS)	Improved/stable	27	14	13		
		Deteriorated	15	5	10	2.2 (0.61–7.7)	0.34
	Mental component summary (MCS)	Improved/stable	26	12	14		
		Deteriorated	16	7	9	1.1 (0.32–4.1)	0.17

*ICI* immune checkpoint inhibittor, *EORTC QLQ-C30* the European organization for research and treatment of cancer quality of life questionnaire - core 30, *FACT-G* functional assessment of cancer therapy-general, *SF-8* 8-item short form survey.

## Discussion

The present study evaluated the time-course changes in the domains and scales assessed using patient-reported measurements in patients with aUC receiving three different ICIs in different settings: second-line or later pembrolizumab, maintenance avelumab, or adjuvant nivolumab. Although more than five years have passed since the first approval of pembrolizumab for aUC in Japan, real-world data on HRQoL in aUC patients treated with ICIs are sparse. Of note, global health status/QoL improved significantly over time in the Pem group, but no significant changes were noted in the other two treatment groups. Our cohort consisted of a heterogeneous population: patients receiving pembrolizumab as a second-line treatment had active disease at baseline, whereas patients receiving maintenance avelumab or adjuvant nivolumab had controlled disease. Recently, we have revealed difference of oncological efficacy between pembrolizumab as second-line setting and avelumab as maintenance setting, following first‑line platinum‑based chemotherapy in patients with aUC^23^. Patients subsequently receiving pembrolizumab manifested significantly higher response rate (14 and 41%, respectively) and longer progression-free survival as compared to those receiving avelumab. Based on this finding, we suppose that higher response rate and following alleviation from cancer-related symptoms might be associated with the result that global health status/QoL improved significantly over time in the Pem group, but no significant changes were noted in the avelumab group. Because the patients in the Nivo group were free from malignant lesions at the time of treatment initiation, toxicities and tumor recurrence can be directly associated with decline in global health status/QoL.

The KEYNOTE-045 trial, in which overall survival (OS) was compared between pembrolizumab and the investigator’s choice of chemotherapy, included pre-specified HRQoL analyses with PROs during treatment^10^. Pembrolizumab prolonged the time to deterioration (TTD) in global health status/QOL scores compared to chemotherapy. Moreover, patients treated with pembrolizumab had better QoL in the EuroQoL five-dimensional questionnaire utility and visual analog scores than those treated with chemotherapy. A subgroup analysis of 52 Japanese patients consisting of 30 treated with pembrolizumab and 22 treated with chemotherapy demonstrated a trend toward a delay in TTD in global health status/QOL score (20 vs. 15 events; hazard ratio [HR], 0.58, 95% confidence interval [CI], 0.29–1.16)^11^. Consistent with the trial evidence, we found significant improvements after the start of pembrolizumab treatment in many domains and subscales (Table 2). The Pem group in our study showed + 1.31 estimated mean increase per month in global health status/QOL score, while + 2.14 least squares mean change was observed from baseline till week 15 in the Japanese cohort in the KEYNOTE-045 trial^12^.

The JAVELIN Bladder 100 trial, which compared OS between maintenance avelumab plus best supportive care (BSC) and BSC alone in patients with aUC without disease progression who received first-line platinum-containing chemotherapy, included HRQoL analysis as a secondary endpoint^12^. The results determined by descriptive analyses and mixed-effect or repeated-measures models of the National Comprehensive Cancer Network/Functional Assessment of Cancer Therapy Bladder Symptom Index-18 (FBlSI-18) and EQ-5D-5 L were statistically similar between the two arms. Moreover, similar TTD (> 3-point decrease from baseline in the FBlSI-18) curves were observed in both arms (HR 1.26, 95% CI 0.90−1.77). Analyses of the Ave group in our study demonstrated that only FWB in the FACT-G assessment deteriorated significantly (-0.82 estimated mean decrease per month) from the start of maintenance avelumab, whereas other domains and scales did not deteriorate during the treatment (Table 2). Based on these data, maintenance avelumab followed by first-line platinum-containing chemotherapy could be administered with a relatively minimal effect on HRQoL, as reported by the patients.

The CheckMate-274 trial, which compared disease-free survival between adjuvant nivolumab and placebo (the maximum treatment duration was one year) after radical surgery in patients with pathologically confirmed MIUC and/or N+, included HRQoL analyses using the EORTC QLQ-C30 and EQ-5D-3L^13^. Overall, no clinically meaningful deterioration in HRQoL was observed in either arm. Adjuvant nivolumab was not inferior to the placebo in terms of changes from baseline for all main outcomes. Particularly for the visual analog scale, adjuvant nivolumab did not reach the median TTD, while it was approximately 58 weeks for placebo (HR: 0.78, 95% CI, 0.61–1.00, *P* < 0.052). Analyses of the Nivo group in our study demonstrated no clinically meaningful deterioration of domains or functional scales, which is consistent with trial evidence. Notably, bodily pain in SF-8 assessment did not change significantly (-0.02 estimated mean decrease per month) from the start of adjuvant nivolumab. There would be significant difference of time-course change in HRQoL between patients with bladder UC undergoing radical cystectomy and those with upper urinary tract UC undergoing radical nephroureterectomy. Of note, adverse events on QoL assessment can be underscored in patients with radical cystectomy, particularly in terms of bowel function^24^. In the Nivo group of this study, only three patients with bladder UC undergoing cystectomy were included, suggesting that comparison between two cohorts was statistically underpowered. Additionally, the CheckMate-274 trial did not conduct comparative analysis of HRQoL score. Further study is required to clarify the change of HRQoL in post-cystectomy patients.

The FACT − Immune Checkpoint Modulator (FACT-ICM) is a 25-item list and one of the first PROs tools, which focus on toxicity subscale for patients treated with ICIs^25^. This subscale was combined with the FACT-G consisting of a 27-item list. Specifically, the FACT-ICM assesses typical symptoms of immunotherapy, and higher scores indicate a higher symptom burden on the patient. Our study included neither PROs evaluation using the FACT-ICM score nor a survival analysis. Schneidewind et al. conducted a prospective observational pilot study to evaluate the HRQoL using the FACT-ICM questionnaire in 14 patients with mUC receiving pembrolizumab^26^. The FACT-G total score remained stable during therapy. Although the symptom burden on patients did not deteriorate significantly over time (*P* = 0.50), patients with a higher symptom burden (FACT-ICM score > 40) had a significantly shorter OS (*P* < 0.001). However, a systematic review and meta-analysis of patients with pan-cancer observed a positive association between immune-related AE (irAE) and favorable clinical outcomes regarding tumor response, progression-free survival, and OS in the ICI treatment setting^27,28^. However, Sanda et al. reported that patients with aUC who had ICI-induced irAEs, especially dermatologic irAEs, had significantly better OS, progression-free survival, and clinical benefit, suggesting that irAEs could serve as predictive markers of a durable response to ICI therapy for aUC^29^. However, there is a lack of evidence regarding the association between irAEs and clinical outcomes in patients with aUC.

It has been suggested that side effects or adverse events caused by cancer therapy would affect HRQoL during treatment. We found a strong association between the occurrence of deterioration of HRQoL TRAEs ≥ grade 2 and several domains and scales such as role functioning, cognitive functioning, SWB, EWB, bodily pain, vitality, and PCS (Table 3). One limitation of our study was that adverse events were recorded by the investigators according to medical charts. Taarnhøj et al. evaluated the usefulness of patient-reported outcomes version of the common terminology criteria for adverse events (PRO-CTCAE)^30^, which consists of 78 symptom items explored by 128 questions, as many symptoms are explored by attributes on frequency, severity, interference with daily activities and/or presence^9^. Spearman’s correlation analysis revealed significant correlations between almost all PRO-CTCAE items and QoL domains (EORTC QLQ-C30 and QLQ-BLM30). Notably, HRQoL with the strongest correlations with the PRO-CTCAE items included emotional, cognitive, and role functions. This evidence suggests that AEs may have a strong impact on physical and psychological issues that should be managed properly.

We are still under development of prediction tool of significant irAE and detection tool of molecular residual disease for patient selection. Lim et al. found that 11 cytokines were significantly upregulated in patients with severe immune-related toxicities at baseline and early during treatment in patients with advanced melanoma treated with combination anti-CTLA-4 and anti-PD-1 immunotherapy^31^. The calculated score based on 11 cytokines including proinflammatory cytokines such as IL1a, IL2, and IFNα2, might help in the early management of severe, potentially life-threatening irAEs. Accumulating evidences support the significant role of liquid biopsies including circulating tumour DNA (ctDNA) as a prognostic and predictive marker, enabling stratifying patients according to individualized risk of tumor progression and recurrence. Much researches regarding usefulness of ctDNA in the clinical management of aUC have been published to date^32,33^. Detectable level of ctDNA before radical cystectomy is associated with higher risk of tumor recurrence and worse disease-free and overall survival after cystectomy for muscle-invasive bladder UC^32^. In addition, ctDNA level after neoadjuvant systemic therapy can predict the pathological response, with persistently detectable ctDNA being associated with residual disease at radical cystectomy^32^. IMvigor010, a phase 3, multicenter, randomized trial evaluated efficacy of adjuvant atezolizumab versus observation in patients with muscle-invasive UC. Subsequent analysis of the data demonstrated that patients who were ctDNA-positive post-cystectomy had improved survivals with atezolizumab compared to observation^34^. This finding inspired the ongoing IMvigor011 trial, which evaluates adjuvant atezolizumab in ctDNA-positive patients undergoing cystectomy^35^. Currently, a new molecular targeting, erdafitinib, have been available for patients with metastatic bladder UC. A cross-sectional case study (NCT06129084) is ongoing to evaluate the diagnostic value of ctDNA testing for FGFR gene mutation in those patients. The investigators are expecting that the blood test will give a more accurate result compared to archival tissue testing. Clinically available tools for selecting patients who should be treated with highly intensified treatment will improve therapeutic index.

Another issue to be discussed is the care and attrition of patients with aUC who discontinue ICI therapy. Morgans et al. ambispectively evaluated real-world treatment patterns and post-ICI PROs in 300 patients receiving ICI therapy^36^. 64% of patients with available PROs data from the EORTC QLQ-C30 experienced cancer-related pain, and 29.6% received an opioid painkiller. Symptoms and caregiver burden are high among real-world patients with aUC after discontinuation of first- or second-line ICI therapy. Patterns in patient care and evidence of HRQoL after ICI therapy and novel agents including enfortumab vedotin require further investigation.

The limitations of this study are as follows: (a) The small-scale and single-center design (only 42 patients overall) is a drawback of this research. The extrapolation of our finding to the Japanese population could be limited. However, the diversity of the questionnaires used by the participants is highly relevant, and it would probably be difficult to include more participants while keeping the same endpoint for a single-centre study.Although several items associated with PROs were evaluated, most did not reach statistical significance, partly because of underpowered samples. (b) This study did not include risk prediction, prevention, or intervention for deterioration of HRQoL during ICI therapy. (c) A possible selection bias cannot be excluded, and the prospective observational design may have resulted in residual confounding bias. (d) We could not compare the PROs data of the three cohorts because the treatment settings were different: second-line or later pembrolizumab after first-line chemotherapy, maintenance avelumab without disease progression with first-line chemotherapy, and adjuvant nivolumab after radical surgery. We did not establish a control arm for comparison purposes.

In conclusion, issues regarding the HRQoL should be addressed during all phases of treatment in patients with UC. When selecting the optimal cancer treatment, multiple aspects should be considered, including oncological outcomes, functional outcomes, and HRQoL. Our findings demonstrated that the majority of patients with aUC who were treated with ICI had a stable HRQoL, which is consistent with evidence from clinical trials. Physical issues and emotional needs are specific to diseases and therapy. For further research, the development of PROs tools for disease- and therapy-specific HRQoL would be of interest so that specific problems in patients can be identified and managed.

## Supplementary Information

Supplementary Figure S1

Supplementary Table S1

## Data Availability

All data generated or analyzed during this study are included in this published article.
