# Supplementary material for: Longitudinal assessment of health-related quality of life in Japanese patients with advanced urothelial carcinoma receiving immune check point inhibitors
Source: Sci Rep. 2024 Oct 4;14:23128. doi: 10.1038/s41598-024-72755-8 (PMC11452380; doi:10.1038/s41598-024-72755-8)
Supplement: Supplementary file 1 — Supplementary Figure S1 [file 41598_2024_72755_MOESM1_ESM.pptx]

## Slide 1
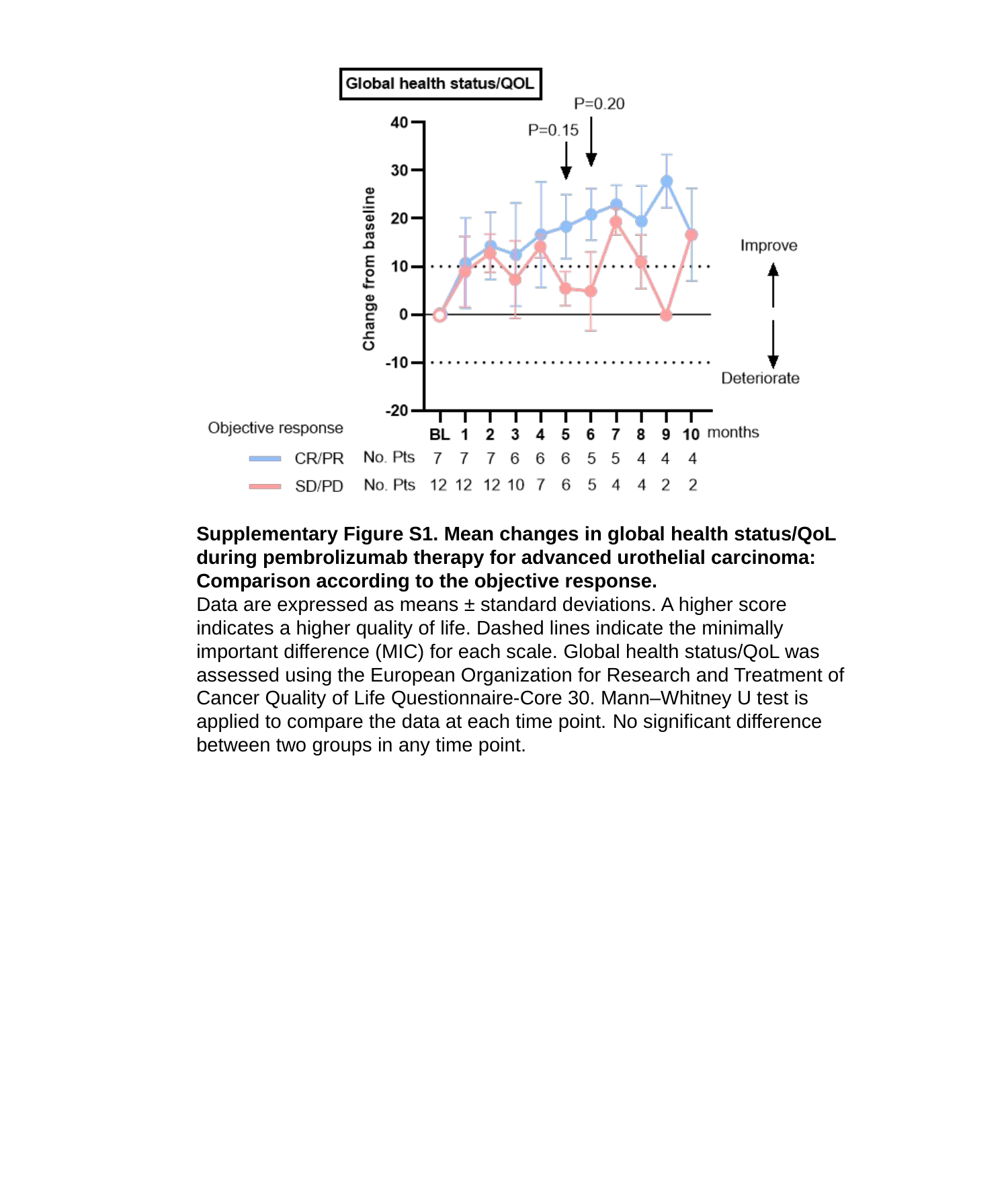

Supplementary Figure S1. Mean changes in global health status/QoL
during pembrolizumab therapy for advanced urothelial carcinoma: Comparison according to the objective response.
Data are expressed as means ± standard deviations. A higher score indicates a higher quality of life. Dashed lines indicate the minimally important difference (MIC) for each scale. Global health status/QoL was assessed using the European Organization for Research and Treatment of Cancer Quality of Life Questionnaire-Core 30. Mann–Whitney U test is applied to compare the data at each time point. No significant difference between two groups in any time point.
