# Supplementary figures and images for: Longitudinal assessment of health-related quality of life in Japanese patients with advanced urothelial carcinoma receiving immune check point inhibitors
Source: Sci Rep. 2024 Oct 4;14:23128. doi: 10.1038/s41598-024-72755-8 (PMC11452380; doi:10.1038/s41598-024-72755-8)

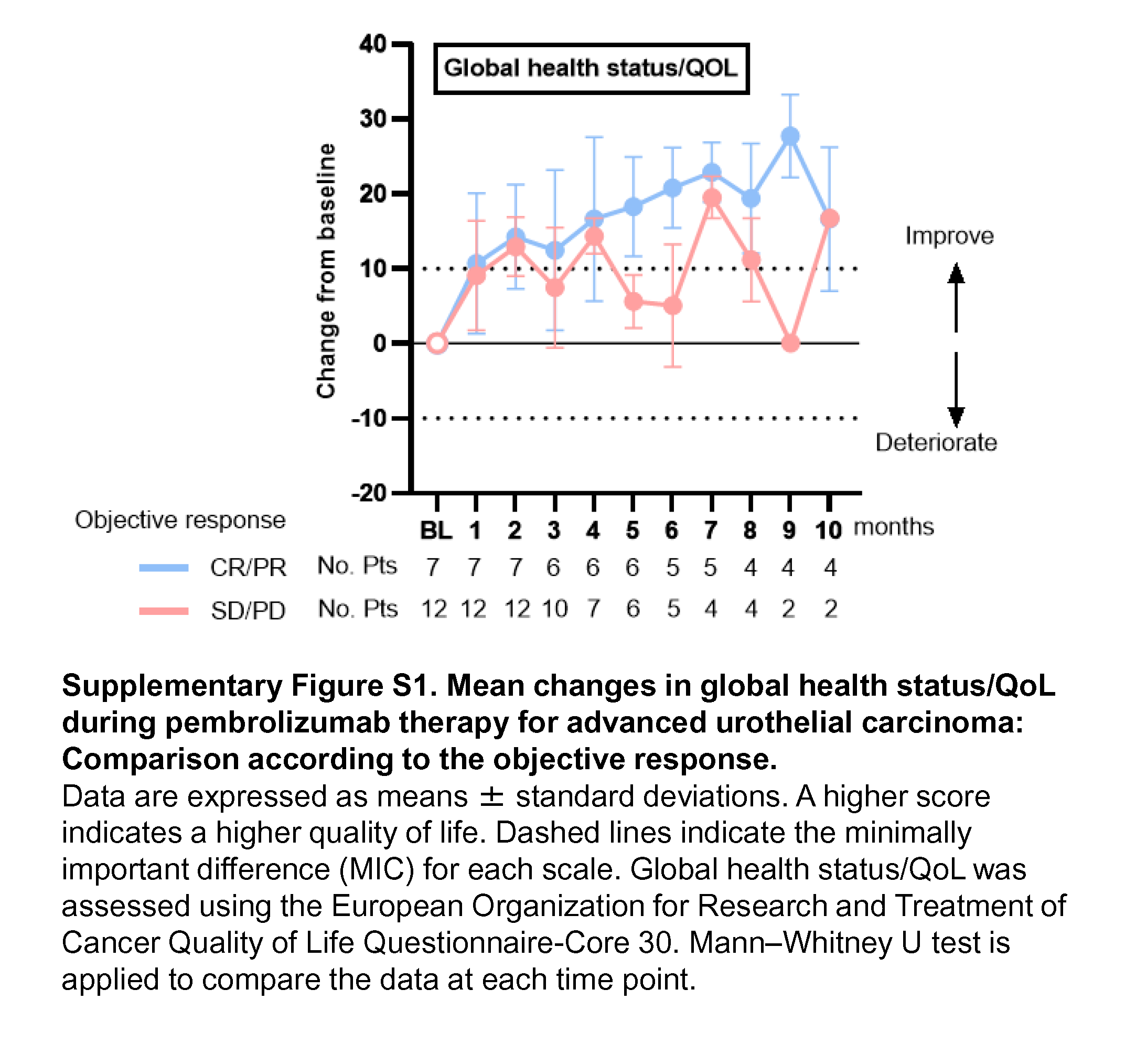

Supplement: Supplementary file 2 — Supplementary Table S1 [file 41598_2024_72755_MOESM2_ESM.tif]
